# (*E*)-(2,4-Dichloro­benzyl­idene)amino cyclo­propane­carboxyl­ate

**DOI:** 10.1107/S1600536812018016

**Published:** 2012-05-02

**Authors:** Mei-Yi Wang, Ya Zhang

**Affiliations:** a College of Chemistry and Chemical Engineering, Beifang University of Nationalities, Yinchuan 750021, People’s Republic of China

## Abstract

In the title compound C_11_H_9_Cl_2_NO_2_, the dihedral angle between the benzene and cyclo­propane ring planes is 89.95 (13)°. The carbon­yl–oxime grouping is almost coplanar with the benzene ring [dihedral angle = 4.08 (6)°]. In the crystal, mol­ecules are linked by C—H⋯O inter­actions into [100] chains.

## Related literature

For further synthetic details, see: Liu *et al.* (2011*b* ▶, 2012 ▶). For related structures, see: Liu & Liu (2011 ▶) Liu *et al.* (2011*d* ▶). For the biological activity of related compounds, see: Liu *et al.* (2010 ▶, 2011*a* ▶,*c* ▶).
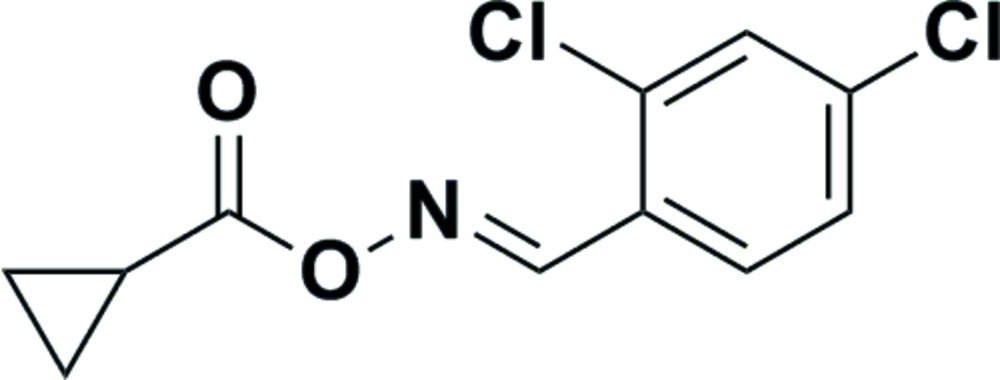


## Experimental

### 

#### Crystal data

C_11_H_9_Cl_2_NO_2_
*M* *_r_* = 258.09
Triclinic, 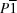

*a* = 6.4381 (13) Å
*b* = 7.6030 (15) Å
*c* = 11.956 (2) Å
α = 94.90 (3)°
β = 100.42 (3)°
γ = 102.70 (3)°
*V* = 556.71 (19) Å^3^
*Z* = 2
Mo *K*α radiation
μ = 0.57 mm^−1^
*T* = 113 K
0.24 × 0.20 × 0.10 mm

#### Data collection

Rigaku Saturn CCD diffractometer
Absorption correction: multi-scan (*CrystalClear*; Rigaku/MSC, 2005 ▶) *T*_min_ = 0.876, *T*_max_ = 0.946
3737 measured reflections
1937 independent reflections
1563 reflections with *I* > 2σ(*I*)
*R*_int_ = 0.030

#### Refinement

*R*[*F* ^2^ > 2σ(*F* ^2^)] = 0.027
*wR*(*F* ^2^) = 0.076
*S* = 1.03
1937 reflections
145 parameters
H-atom parameters constrained
Δρ_max_ = 0.24 e Å^−3^
Δρ_min_ = −0.21 e Å^−3^

### 

Data collection: *CrystalClear* (Rigaku/MSC, 2005 ▶); cell refinement: *CrystalClear*; data reduction: *CrystalClear*; program(s) used to solve structure: *SHELXS97* (Sheldrick, 2008 ▶); program(s) used to refine structure: *SHELXL97* (Sheldrick, 2008 ▶); molecular graphics: *SHELXTL* (Sheldrick, 2008 ▶); software used to prepare material for publication: *SHELXL97*.

## Supplementary Material

Crystal structure: contains datablock(s) global, I. DOI: 10.1107/S1600536812018016/hb6735sup1.cif

Structure factors: contains datablock(s) I. DOI: 10.1107/S1600536812018016/hb6735Isup2.hkl

Supplementary material file. DOI: 10.1107/S1600536812018016/hb6735Isup3.cml

Additional supplementary materials:  crystallographic information; 3D view; checkCIF report

## Figures and Tables

**Table 1:** Hydrogen-bond geometry (Å, °)

*D*—H⋯*A*	*D*—H	H⋯*A*	*D*⋯*A*	*D*—H⋯*A*
C1—H1*B*⋯O1^i^	0.97	2.57	3.5008 (19)	161

Symmetry code: (i) 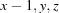.

**Fig. 1.**
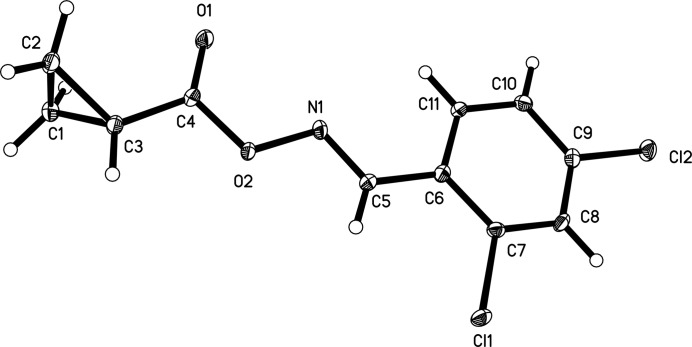
The molecular structure of (I). Displacement ellipsoids are drawn at the 30% probability level.

## supplementary crystallographic information

### Experimental

Dropwised the cyclopropanecarbonyl chloride to 2,4-dichlorobenzaldehyde oxime (7.50 mmol in 25 ml THF) and 7.5 mmol Et3N, then vigorously stirred at ambient temperature for overnight. The corresponding product precipitated immediately. Compound was dissolved in hot alcohol and the resulting solution was allowed to stand in air at room temperature to give colourless blocks of the title compound.

### Refinement

All the H atoms were positioned geometrically (C—H = 0.93–0.97 Å) and refined as riding with *U*_iso_(H) = 1.2*U*_eq_(C) or 1.5*U*_eq_(methyl C).

### Crystal data

**Table:**

C_11_H_9_Cl_2_NO_2_	*Z* = 2
*M**_r_* = 258.09	*F*(000) = 264
Triclinic, *P*1	*D*_x_ = 1.540 Mg m^−^^3^
*a* = 6.4381 (13) Å	Mo *K*α radiation, λ = 0.71073 Å
*b* = 7.6030 (15) Å	Cell parameters from 1887 reflections
*c* = 11.956 (2) Å	θ = 1.8–27.9°
α = 94.90 (3)°	µ = 0.57 mm^−^^1^
β = 100.42 (3)°	*T* = 113 K
γ = 102.70 (3)°	Block, coloress
*V* = 556.71 (19) Å^3^	0.24 × 0.20 × 0.10 mm

### Data collection

**Table:**

Rigaku Saturn CCD diffractometer	1937 independent reflections
Radiation source: rotating anode	1563 reflections with *I* > 2σ(*I*)
Confocal monochromator	*R*_int_ = 0.030
ω scans	θ_max_ = 25.0°, θ_min_ = 1.8°
Absorption correction: multi-scan (*CrystalClear*; Rigaku/MSC, 2005)	*h* = −7→7
*T*_min_ = 0.876, *T*_max_ = 0.946	*k* = −9→6
3737 measured reflections	*l* = −14→14

### Refinement

**Table:**

Refinement on *F*^2^	Primary atom site location: structure-invariant direct methods
Least-squares matrix: full	Secondary atom site location: difference Fourier map
*R*[*F*^2^ > 2σ(*F*^2^)] = 0.027	Hydrogen site location: inferred from neighbouring sites
*wR*(*F*^2^) = 0.076	H-atom parameters constrained
*S* = 1.03	*w* = 1/[σ^2^(*F*_o_^2^) + (0.0407*P*)^2^] where *P* = (*F*_o_^2^ + 2*F*_c_^2^)/3
1937 reflections	(Δ/σ)_max_ = 0.001
145 parameters	Δρ_max_ = 0.24 e Å^−^^3^
0 restraints	Δρ_min_ = −0.21 e Å^−^^3^

### Special details

**Table:**

Geometry. All e.s.d.'s (except the e.s.d. in the dihedral angle between two l.s. planes) are estimated using the full covariance matrix. The cell e.s.d.'s are taken into account individually in the estimation of e.s.d.'s in distances, angles and torsion angles; correlations between e.s.d.'s in cell parameters are only used when they are defined by crystal symmetry. An approximate (isotropic) treatment of cell e.s.d.'s is used for estimating e.s.d.'s involving l.s. planes.
Refinement. Refinement of *F*^2^ against ALL reflections. The weighted *R*-factor *wR* and goodness of fit *S* are based on *F*^2^, conventional *R*-factors *R* are based on *F*, with *F* set to zero for negative *F*^2^. The threshold expression of *F*^2^ > σ(*F*^2^) is used only for calculating *R*-factors(gt) *etc*. and is not relevant to the choice of reflections for refinement. *R*-factors based on *F*^2^ are statistically about twice as large as those based on *F*, and *R*- factors based on ALL data will be even larger.

### Fractional atomic coordinates and isotropic or equivalent isotropic displacement parameters (Å^2^)

**Table:**

	*x*	*y*	*z*	*U*_iso_*/*U*_eq_	
Cl1	0.32359 (6)	0.70035 (5)	0.47089 (3)	0.02317 (14)	
Cl2	1.15092 (6)	0.98054 (6)	0.68823 (4)	0.02531 (14)	
O1	0.08882 (17)	0.30779 (16)	0.94813 (9)	0.0263 (3)	
O2	0.00692 (16)	0.37138 (15)	0.76492 (9)	0.0186 (3)	
N1	0.2306 (2)	0.46280 (18)	0.78101 (11)	0.0202 (3)	
C1	−0.3868 (2)	0.2548 (2)	0.94150 (14)	0.0217 (4)	
H1A	−0.2971	0.3293	1.0106	0.026*	
H1B	−0.5348	0.2689	0.9234	0.026*	
C2	−0.3504 (3)	0.0724 (2)	0.91336 (14)	0.0208 (4)	
H2A	−0.4764	−0.0249	0.8783	0.025*	
H2B	−0.2388	0.0354	0.9655	0.025*	
C3	−0.2777 (2)	0.2198 (2)	0.84222 (14)	0.0198 (4)	
H3	−0.3629	0.2113	0.7644	0.024*	
C4	−0.0426 (2)	0.3004 (2)	0.86199 (13)	0.0177 (4)	
C5	0.2637 (2)	0.5304 (2)	0.69054 (14)	0.0177 (4)	
H5	0.1511	0.5129	0.6268	0.021*	
C6	0.4823 (2)	0.6365 (2)	0.68810 (14)	0.0164 (4)	
C7	0.5265 (3)	0.7218 (2)	0.59348 (13)	0.0168 (4)	
C8	0.7305 (2)	0.8271 (2)	0.59131 (13)	0.0188 (4)	
H8	0.7566	0.8836	0.5275	0.023*	
C9	0.8947 (2)	0.8453 (2)	0.68750 (14)	0.0187 (4)	
C10	0.8595 (3)	0.7612 (2)	0.78232 (14)	0.0196 (4)	
H10	0.9725	0.7736	0.8453	0.024*	
C11	0.6539 (2)	0.6579 (2)	0.78256 (14)	0.0177 (4)	
H11	0.6293	0.6016	0.8466	0.021*	

### Atomic displacement parameters (Å^2^)

**Table:**

	*U*^11^	*U*^22^	*U*^33^	*U*^12^	*U*^13^	*U*^23^
Cl1	0.0227 (2)	0.0314 (3)	0.0143 (2)	0.00420 (18)	0.00140 (17)	0.00786 (18)
Cl2	0.0192 (2)	0.0275 (2)	0.0273 (3)	−0.00120 (17)	0.00716 (17)	0.00581 (18)
O1	0.0182 (6)	0.0425 (8)	0.0166 (7)	0.0021 (5)	0.0021 (5)	0.0121 (6)
O2	0.0123 (5)	0.0267 (6)	0.0165 (6)	0.0012 (5)	0.0038 (4)	0.0082 (5)
N1	0.0117 (6)	0.0267 (8)	0.0213 (8)	0.0005 (6)	0.0039 (5)	0.0072 (6)
C1	0.0179 (8)	0.0266 (9)	0.0221 (10)	0.0051 (7)	0.0073 (7)	0.0061 (7)
C2	0.0191 (8)	0.0226 (9)	0.0198 (9)	0.0004 (7)	0.0059 (7)	0.0054 (7)
C3	0.0151 (8)	0.0264 (9)	0.0160 (9)	0.0002 (7)	0.0027 (7)	0.0059 (7)
C4	0.0187 (8)	0.0201 (8)	0.0162 (9)	0.0045 (7)	0.0070 (7)	0.0059 (7)
C5	0.0178 (8)	0.0218 (9)	0.0145 (9)	0.0048 (7)	0.0041 (7)	0.0056 (7)
C6	0.0184 (8)	0.0158 (8)	0.0162 (9)	0.0042 (6)	0.0061 (7)	0.0031 (6)
C7	0.0185 (8)	0.0193 (8)	0.0134 (9)	0.0070 (6)	0.0019 (6)	0.0024 (6)
C8	0.0244 (9)	0.0189 (9)	0.0165 (10)	0.0057 (7)	0.0100 (7)	0.0071 (7)
C9	0.0181 (8)	0.0170 (8)	0.0223 (9)	0.0038 (7)	0.0082 (7)	0.0024 (7)
C10	0.0189 (8)	0.0226 (9)	0.0183 (9)	0.0077 (7)	0.0022 (7)	0.0040 (7)
C11	0.0194 (8)	0.0206 (9)	0.0166 (9)	0.0079 (7)	0.0065 (7)	0.0073 (7)

### Geometric parameters (Å, º)

**Table:**

Cl1—C7	1.7477 (16)	C3—C4	1.470 (2)
Cl2—C9	1.7394 (16)	C3—H3	0.9800
O1—C4	1.1976 (18)	C5—C6	1.467 (2)
O2—C4	1.3800 (19)	C5—H5	0.9300
O2—N1	1.4253 (16)	C6—C7	1.394 (2)
N1—C5	1.269 (2)	C6—C11	1.401 (2)
C1—C2	1.478 (2)	C7—C8	1.385 (2)
C1—C3	1.517 (2)	C8—C9	1.389 (2)
C1—H1A	0.9700	C8—H8	0.9300
C1—H1B	0.9700	C9—C10	1.378 (2)
C2—C3	1.508 (2)	C10—C11	1.383 (2)
C2—H2A	0.9700	C10—H10	0.9300
C2—H2B	0.9700	C11—H11	0.9300
			
C4—O2—N1	112.18 (11)	O2—C4—C3	109.31 (12)
C5—N1—O2	109.05 (12)	N1—C5—C6	118.94 (14)
C2—C1—C3	60.45 (11)	N1—C5—H5	120.5
C2—C1—H1A	117.7	C6—C5—H5	120.5
C3—C1—H1A	117.7	C7—C6—C11	117.60 (14)
C2—C1—H1B	117.7	C7—C6—C5	121.71 (14)
C3—C1—H1B	117.7	C11—C6—C5	120.69 (15)
H1A—C1—H1B	114.8	C8—C7—C6	122.37 (14)
C1—C2—C3	61.08 (11)	C8—C7—Cl1	116.74 (13)
C1—C2—H2A	117.7	C6—C7—Cl1	120.89 (12)
C3—C2—H2A	117.7	C7—C8—C9	117.79 (15)
C1—C2—H2B	117.7	C7—C8—H8	121.1
C3—C2—H2B	117.7	C9—C8—H8	121.1
H2A—C2—H2B	114.8	C10—C9—C8	121.89 (15)
C4—C3—C2	116.62 (14)	C10—C9—Cl2	119.36 (12)
C4—C3—C1	115.92 (13)	C8—C9—Cl2	118.75 (13)
C2—C3—C1	58.47 (11)	C9—C10—C11	119.14 (14)
C4—C3—H3	117.5	C9—C10—H10	120.4
C2—C3—H3	117.5	C11—C10—H10	120.4
C1—C3—H3	117.5	C10—C11—C6	121.20 (15)
O1—C4—O2	123.97 (14)	C10—C11—H11	119.4
O1—C4—C3	126.72 (15)	C6—C11—H11	119.4
			
C4—O2—N1—C5	−177.60 (13)	C5—C6—C7—C8	177.96 (16)
C1—C2—C3—C4	105.44 (16)	C11—C6—C7—Cl1	179.11 (13)
C2—C1—C3—C4	−106.64 (16)	C5—C6—C7—Cl1	−2.0 (2)
N1—O2—C4—O1	−3.1 (2)	C6—C7—C8—C9	0.4 (3)
N1—O2—C4—C3	176.04 (13)	Cl1—C7—C8—C9	−179.65 (12)
C2—C3—C4—O1	−25.6 (3)	C7—C8—C9—C10	0.6 (3)
C1—C3—C4—O1	40.4 (2)	C7—C8—C9—Cl2	−178.69 (13)
C2—C3—C4—O2	155.33 (15)	C8—C9—C10—C11	−1.1 (3)
C1—C3—C4—O2	−138.66 (14)	Cl2—C9—C10—C11	178.24 (13)
O2—N1—C5—C6	178.12 (13)	C9—C10—C11—C6	0.5 (3)
N1—C5—C6—C7	−176.72 (16)	C7—C6—C11—C10	0.5 (2)
N1—C5—C6—C11	2.2 (3)	C5—C6—C11—C10	−178.44 (16)
C11—C6—C7—C8	−1.0 (3)		

### Hydrogen-bond geometry (Å, º)

**Table:**

*D*—H···*A*	*D*—H	H···*A*	*D*···*A*	*D*—H···*A*
C1—H1*B*···O1^i^	0.97	2.57	3.5008 (19)	161

Symmetry code: (i) *x*−1, *y*, *z*.
